# Four-Parameter FluoroSpot Assay Reveals That the Varicella Zoster Virus Elicits a Robust Memory T Cell IL-10 Response throughout Childhood

**DOI:** 10.1128/jvi.01310-22

**Published:** 2022-10-31

**Authors:** Anna Nilsson, Anna Höbinger, Peter Jahnmatz, Eva Tiselius, Åsa Laestadius, Erik Melén, Sophia Björkander, Shanie Saghafian-Hedengren

**Affiliations:** a Department of Women’s and Children’s Health, Division of Paediatric Oncology and Paediatric Surgery, Karolinska Institute t, Stockholm, Sweden; b Division of Infectious Diseases, Department of Medicine Solna, Karolinska Institute t and Center for Molecular Medicine, Stockholm, Sweden; c Mabtech AB, Nacka Strand, Sweden; d Department of Women’s and Children’s Health, Karolinska Institute t, Stockholm, Sweden; e Department of Clinical Science and Education, Södersjukhuset, Karolinska Institute t, Stockholm, Sweden; University of Kentucky College of Medicine

**Keywords:** IL-10, children, human herpesviruses, memory T cells

## Abstract

During childhood, the composition and function of the T cell compartment undergoes significant changes. In healthy individuals, primary infection with herpesviruses is followed by latency, and occasional subclinical reactivation ensures transmission and contributes to an emerging pool of memory T cells. In immunocompromised individuals, herpesviruses can be life threatening. However, knowledge about the spectrum of virus-specific cytokine responses is limited. Here, we investigated peripheral blood mononuclear cells (PBMCs) from children with differential carrier statuses for cytomegalovirus (CMV), Epstein-Barr virus (EBV), and varicella zoster virus (VZV) (*n *= 32, age 1 to 17 years). We examined memory T cell subsets as well as IFN-γ-, IL-10-, IL-17A-, and IL-22-producing T cells after polyclonal activation or stimulation with viral peptides using flow cytometry and a 4-parameter FluoroSpot assay. Age and herpesvirus carriage influenced the size of the memory T cell subsets. A positive association between age and the number of IFN-γ-, IL-17A- and IL-22-producing T cells was found following polyclonal activation. For CMV, age was positively associated with IL-17A spot-forming cells (SFC), while for VZV, age was negatively associated with IL-22 and positively associated with IFN-γ SFC. Upon activation with CMV, VZV, and EBV peptides, IFN-γ SFCs dominated. Notably, VZV responses were characterized by a higher IL-10 SFC population compared to both CMV and EBV. Our findings suggest that cytokine responses vary across herpesvirus-type-specific memory T cells and may more adequately reflect their composition. An observed deviation between polyclonal and herpesvirus-specific T cell cytokine responses in children needs to be considered when interpreting the associations between herpesvirus carrier status and bulk T cell reactivity. In summary, these findings may have implications for the treatment of immunocompromised patients.

**IMPORTANCE** Infection with herpesviruses accounts for 35 to 40 billion human cases worldwide. Despite this, little is known about how herpesviruses shape the immune system in the asymptomatic carrier. Particularly in children, primary infection is connected to no or mild symptoms ahead of latency for life. Most research on cellular responses against herpesviruses focuses on inflammatory cytokines associated with antiproliferative and antitumor mechanisms and not the spectrum of cytokine responses in healthy humans. This study investigated four divergent cytokine-producing T cell responses to herpesviruses, reflecting different immunological functions. Three common childhood herpesviruses were selected: Epstein-Barr virus, cytomegalovirus, and varicella-zoster virus. Curiously, not all viruses induced the same pattern of cytokines. Varicella-zoster responses were characterized by IL-10, which is considered regulatory. Besides broadening understanding of responses to herpesviruses, our results raise the possibility that reactivation of varicella-zoster may be counterproductive in cancer treatment through the action of IL-10-producing T-cells.

## INTRODUCTION

Systems immunology studies assessing the phenotypes of peripheral blood cells from large cohorts have provided evidence that age, environment, and chronic viral infections contribute greatly to variations in immune composition and function (1–5). The T cell compartment undergoes significant changes during the early years of life. As a result of the marked expansion of recent thymic emigrants and naive T cells, absolute T cell numbers increase rapidly after birth. This is followed by a gradual decrease in T cell numbers during the first years of life to progressively match adult levels. Due to a lack of previous exposure to antigens, a high susceptibility to pathogens occurs following birth, during infancy and early childhood. As a result of exposure to various new antigens, the generation of memory T cell populations is most intense during infancy, childhood, and adolescence, followed by a plateau phase maintained through homeostasis during adulthood (6). Regulatory T cells, on the other hand, are highly abundant early in life (reference 7 and references within). Naïve T cells (TN) progressively differentiate into various memory T cell subsets following antigen priming. Eventually, memory T cells differentiate into terminally differentiated effector T cells (TEFF), which are paralleled by loss or gain of specific tissue-homing and functional features. Memory T cells are abundant in the blood of adult humans and can be divided based on their phenotype into central memory (TCM), effector memory (TEM), and stem-central memory (TSCM) T cells. TSCM cells are a relatively rare subset with high proliferative and self-renewal capabilities without effector functions (8). TCM and TEM cells, on the other hand, can produce effector cytokines after activation. While TCM cells exhibit lymphoid-homing profiles and high proliferative potential, TEM cells have less proliferative capacity and preferentially home to peripheral tissues, although they elicit more rapid effector functions and cytokine release (9, 10).

Herpesvirus infections are abundant in human populations and have a great impact on shaping the composition and function of the T cell compartment in children and adults (11 to 14). Following a self-limiting primary infection in a healthy host, herpesviruses persist for life by becoming latent, with intermittent reactivation to ensure viral spread. There are eight known human herpesviruses, and most of us carry multiple of these at the same time. Most immunocompetent individuals who carry herpesviruses are asymptomatic. However, in immunosuppressed individuals, reactivation of herpesviruses can become life-threatening. Herpesvirus latency is a period of ongoing interaction between the virus and its host, resulting in a large pool of highly functional memory CD4^+^ and CD8^+^ T cells that are maintained for life to ensure viral control (15). Three of the most studied human herpesviruses are cytomegalovirus (CMV), Epstein-Barr virus (EBV), and varicella-zoster virus (VZV). Approximately 10 to 20% of circulating memory CD4^+^ and CD8^+^ T cells in healthy adults are specific for cytomegalovirus CMV antigens (16), 0.1 to 1.3% memory CD4^+^ and 5 to 10% memory CD8^+^ T cells for EBV antigens (17, 18), and up to 1.8% of interferon gamma (IFN-g)-producing memory T cells are reactive against VZV antigens (19). Contrary to the clear role for CD8^+^ T cells in controlling CMV and EBV infection, several lines of evidence suggest that cytotoxic T cell responses may not be as crucial as CD4^+^ T cell responses in the control of VZV infection (20).

Despite intense research on immune responses against herpesviruses, knowledge about T cell responses during childhood and adolescence in otherwise healthy individuals is still limited. In addition, most studies on T cell functionality have focused on the type-1 cytokines IFN-γ and tumor necrosis factor (TNF) in immunocompromised patients, thus overlooking the broader spectrum of cytokine-producing T cell subsets, which may more adequately reflect herpesvirus-driven maturation of T cell responses. To evaluate this, we used peripheral blood mononuclear cells (PBMCs) from CMV, EBV, and/or VZV latently infected children (age 1 to 17 years), with or without pediatric rheumatic diseases (PRD), to assess circulating memory T cell subsets *ex vivo* and the numbers of IFN-γ-, interleukin 10 (IL-10)-, IL-17A-, and IL-22-producing cells after *in vitro* stimulation with CMV, EBV, or VZV.

## RESULTS

### Impact of age, autoimmune disease, treatment, and herpesvirus type on blood T cell subsets.

**FIG 1:**
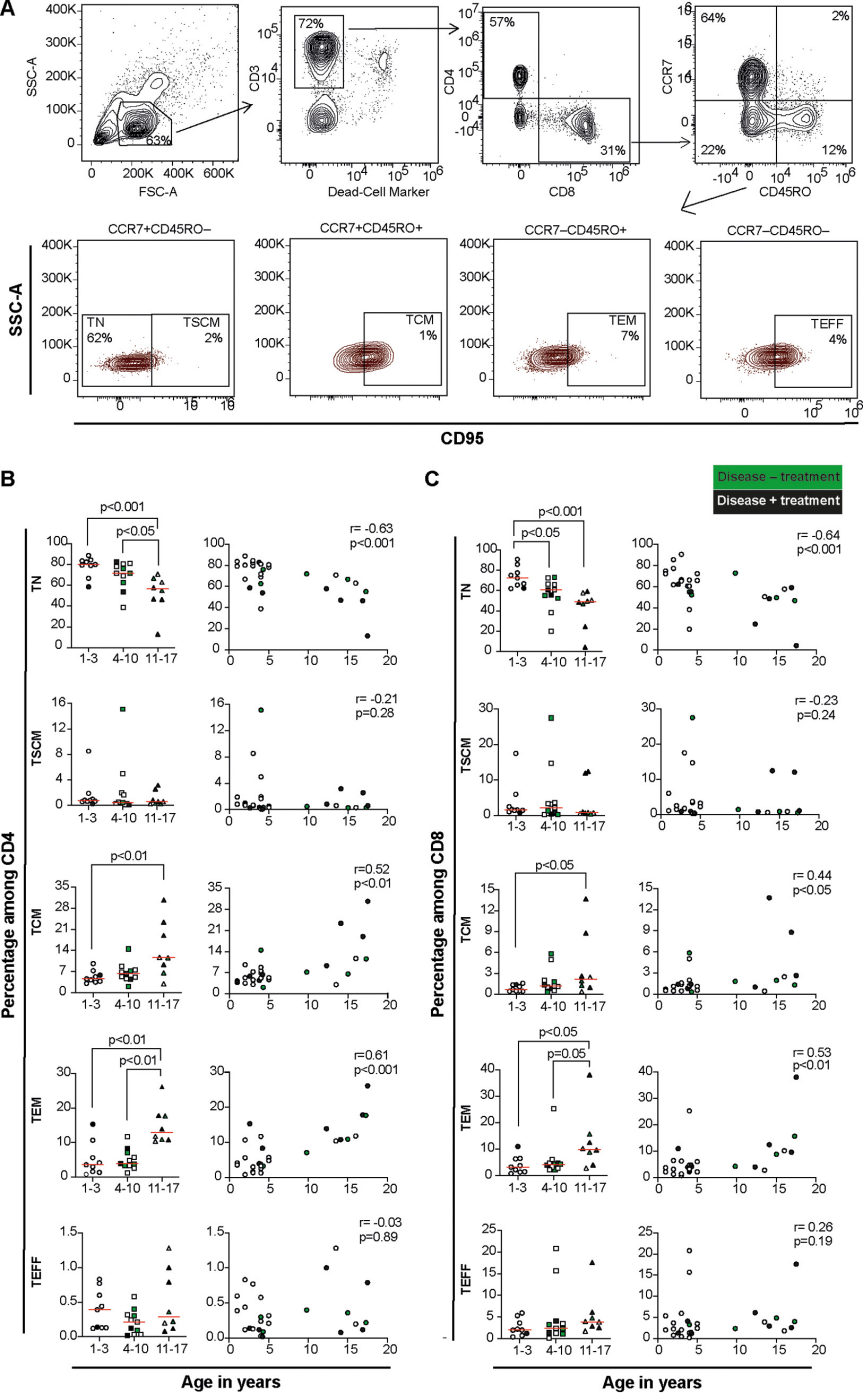
Relationship between age and T cell populations in healthy children and children with autoimmune diseases. (A) Representative gating strategy of T cell populations from peripheral blood mononuclear cells. (B, C) Proportions of memory T cell subsets among bulk CD4^+^ T cells (B, left-hand graphs) and CD8^+^ T cells (C, left-hand graphs) and correlations between age and proportions of memory T cell subsets among CD4^+^ T cells (B, right-hand graphs) and CD8^+^ T cells (C, right-hand graphs). Kruskal-Wallis ANOVA was applied to test the statistical differences among the three age categories, and statistical differences were considered if *P* values were at <0.05 after Dunn’s correction for multiple comparisons. Correlations were investigated using Spearman rank correlation. *n* = 28 among whom a subset (*n *= 12) had pediatric rheumatic disease with treatment (*n *= 7, filled black symbols) or without treatment (*n *= 5, filled green symbols).

The relationship between age and the composition of five established blood T cell phenotypes (21) was first examined for the different age groups (Fig. 1A). The proportions of TN in both CD4^+^ and CD8^+^ T cell lineages generally decreased with increasing age and were significantly lower in the oldest age category of children compared to the youngest (Fig. 1B and C, *P < *0.01 and *P < *0.001, respectively), which was supported when considering age as a continuous variable (Fig. 1B and C). The decrease in the TN T cell pool was paralleled with increasing proportions of TCM and TEM T cells in both CD4 and CD8 T cell lineages, which was most notable when the group of youngest children was compared with the oldest group (left-hand panels, Fig. 1B and C, *P < *0.01 TCM and TEM, and *P < *0.05 TCM and TEM, respectively). Similarly, a positive relationship between age and emergence of TCM and TEM T cell populations was also observed in the correlation analysis (right-hand panels, Fig. 1B and C). There were no significant differences in the proportions of TSCM and TEFF T cells (Fig. 1B and C) or of CD3^+^, CD3^+^CD4^+^, or CD3^+^CD8^+^ T cells (Fig. S1 in the supplemental material).

To understand whether autoimmune disease and/or subsequent treatment impacted the composition of peripheral blood T cell subsets, quantile regression analysis, including disease, treatment, and age as covariates, was performed (Table 1). Whereas disease did not significantly impact any of the examined T cell subsets, the size of one subset, TN CD4^+^ T cells, was negatively associated with treatment (adjusted coefficient, Coef^adj^ = −19.0 and *P = *0.003). Age had the greatest impact on CD4^+^ T cell composition (TN: Coef^adj^ −0.9, *P = *0.028; TCM: Coef^adj^ 0.5, *P = *0.028; TEM: Coef^adj^ 0.7, *P = *0.002) (Table 1). Notably, in the CD8^+^ T cell lineage, age significantly affected only TEM T cells (Coef^adj^ 0.5, *P = *0.033) (Table 1).

**TABLE 1:** Median quantile regression results from T cell phenotypes and clinical parameters^*a*^

	Disease				Treatment				Age			
Cell variable	Coef (CI_L_, CI_U_)	*P*	Coef (CI_L_, CI_U_)^adj^	*P* ^adj^	Coef (CI_L_, CI_U_)	*P*	Coef^adj^ (CI_L_, CI_U_)	*P* ^adj^	Coef (CI_L_, CI_U_)	*P*	Coef^adj^ (CI_L_, CI_U_)	*P* ^adj^
%CD3	3.8 (−9.9, 17.5)	0.575	4.0 (−17.2, 25.3)	0.698	−3.5 (−18.5, 11.5)	0.635	−4.5 (−26.9, 17.8)	0.679	0.2 (−1.0, 1.4)	0.770	−0.1 (−1.6, 1.4)	0.882
%CD4	0.3 (−13.2, 13.8)	0.964	0.1 (−19.5, 19.8)	0.988	−5.8 (−18.9, 7.3)	0.371	−4.3 (−25.0, 16.4)	0.670	−0.2 (−0.3, 0.8)	0.677	−0.2 (−1.6, 1.2)	0.751
%CD8	−1.1 (−10.3, 8.1)	0.807	2.8 (−10.9, 16.5)	0.676	−2.7 (−11.2, 5.8)	0.519	−5.4 (−19.8, 9.0)	0.446	0.2 (−0.6, 1.0)	0.577	0.0 (−1.0, 1.0)	1.000
%CD4 TN	−21.3 (−33.8 to 8.8)	**0.002** ^*b*^	−2.4 (−13.4, 8.6)	0.661	−22.2 (−34.6 to 9.8)	**0.001**	−19.0 (−30.6 to 7.4)	**0.003**	−1.6 (−2.7 to 0.6)	**0.004**	−0.9 (−1.6 to 0.1)	**0.028**
%CD4 TSCM	−0.3 (−1.5, 0.9)	0.593	−0.2 (−4.0, 3.5)	0.906	−0.0 (−1.3, 1.2)	0.949	0.3 (−3.7, 4.2)	0.879	−0.0 (−0.1, 0.1)	0.892	−0.0 (−0.3, 0.2)	0.897
%CD4 TCM	4.0 (−1.3, 9.3)	0.130	−1.2 (−7.9, 5.4)	0.703	4.0 (−2.3, 10.3)	0.206	2.4 (−4.6, 9.4)	0.484	0.5 (0.0, 0.9)	**0.042**	0.5 (0.1, 1.0)	**0.028**
%CD4 TEM	6.8 (−0.1, 13.6)	**0.054**	−0.6 (−6.6, 5.5)	0.851	9.8 (3.2, 16.3)	**0.005**	5.0 (−1.3, 11.4)	0.115	0.7 (0.4, 1.0)	**0.000**	0.7 (0.3, 1.1)	**0.002**
%CD4 TEFF	−0.1 (−0.4, 0.2)	0.512	−0.0 (−0.5, 0.5)	0.938	−0.2 (−0.6, 0.2)	0.335	−0.2 (−0.7, 0.4)	0.508	−0.0 (−0.0, 0.0)	0.921	0.0 (0.0, 0.0)	1.000
%CD8 TN	−13.6 (−26.7 to 0.5)	**0.042**	−10.0 (−27.8, 7.8)	0.259	−10.0 (−29.6, 9.6)	0.305	−0.6 (−19.3, 18.2)	0.948	−1.3 (−2.7, 0.1)	0.070	−0.7 (−2.0, 0.5)	0.245
%CD8 TSCM	−1.3 (−6.2, 3.5)	0.575	−1.5 (−10.4, 7.4)	0.727	−0.7 (−5.5, 4.2)	0.772	0.2 (−9.2, 9.6)	0.965	−0.1 (−0.5, 0.3)	0.730	0.0 (−0.6, 0.6)	0.962
%CD8 TCM	0.7 (−1.1, 2.5)	0.429	−0.1 (−3.3, 3.1)	0.957	0.3 (−1.9, 2.5)	0.779	0.5 (−2.9, 3.8)	0.781	0.1 (−0.0, 0.3)	0.152	0.1 (−0.1, 0.3)	0.401
%CD8 TEM	5.6 (1.6, 9.6)	**0.008**	−0.8 (−7.8, 6.2)	0.818	5.9 (0.9, 10.9)	**0.023**	1.7 (−5.7, 9.0)	0.641	0.4 (0.1, 0.8)	**0.012**	0.5 (0.0, 1.0)	**0.033**
%CD8 TEFF	0.9 (−1.9, 3.6)	0.524	−0.4 (−4.5, 3.6)	0.824	0.5 (−2.5, 3.6)	0.722	−0.6 (−4.8, 3.6)	0.773	0.1 (−0.1, 0.4)	0.296	0.1 (−0.1, 0.4)	0.299

a Coef (CI_L_, CI_U_), coefficient with 95% confidence intervals (L = lower, U = upper); ^adj^, model includes the covariates disease and treatment as binary variables and age as continuous variable.

b Bold numbers indicate significant values.

**FIG 2:**
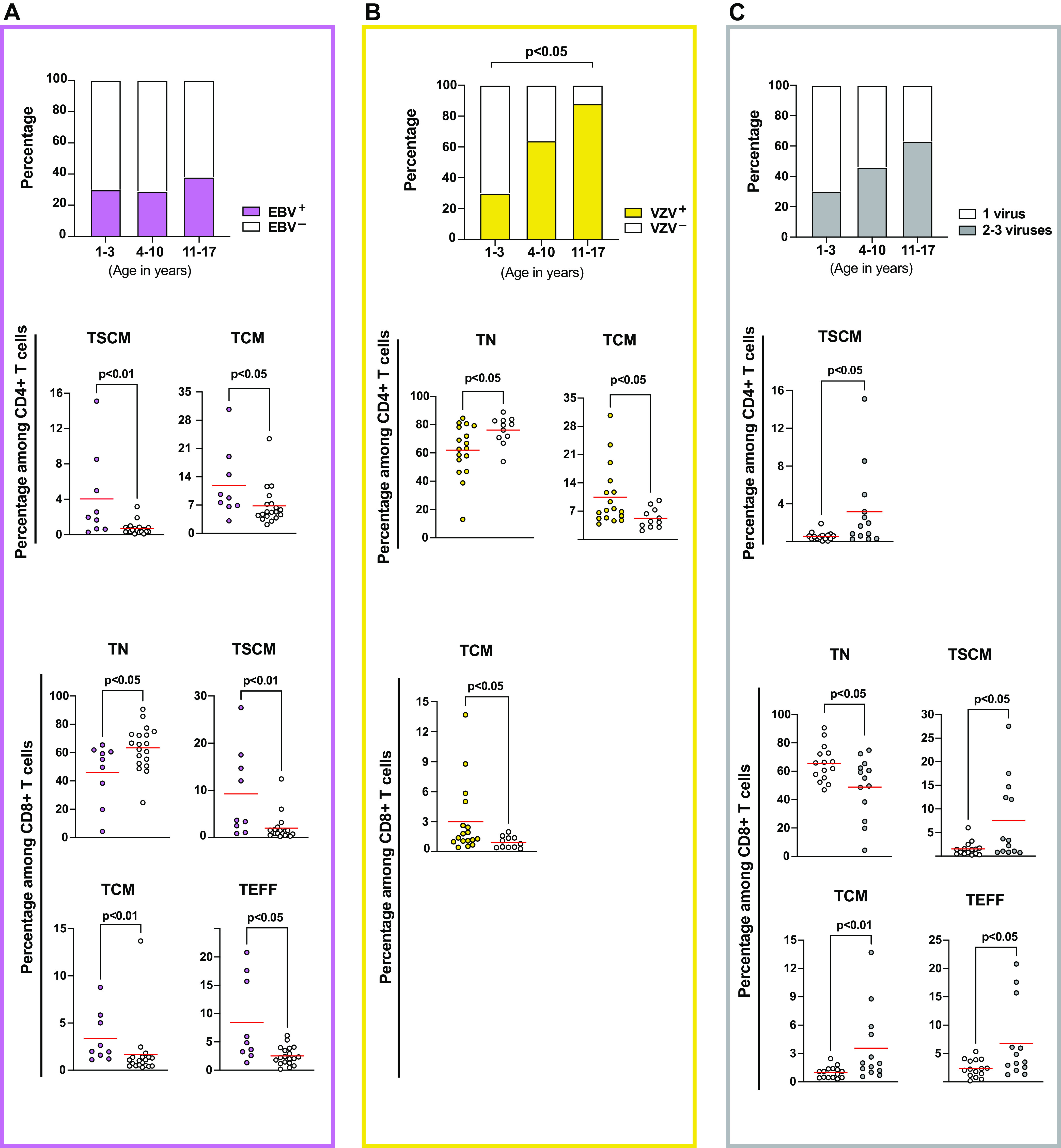
Impact of and herpesvirus carrier status on the size of peripheral blood T cell populations. The proportion of (A) EBV-infected and (B) VZV-infected children and (C) the proportion of children infected with more than one of the investigated viruses within each indicated age group. The percentages of different T cell subsets based on differential serostatus for EBV (A, *n *= 9, filled pink symbols), VZV (B, *n *= 17, filled yellow symbols), or coinfection with these herpesviruses (C, *n *= 13, filled gray symbols), displaying that the size of naive and memory T cell populations among bulk CD4^+^ T cells and CD8^+^ T cells may vary. The Mann-Whitney U test was used to compare 2 independent groups. Kruskal-Wallis ANOVA was applied to test the statistical differences between the three age categories, and statistical differences were considered if *P* values were at <0.05 after Dunn’s correction for multiple comparisons.

We next examined the size of the T cell subsets in relation to the presence or absence of latent infection with EBV, CMV, and VZV (visualized as diagrams for EBV and VZV in Fig. 2; complete data can be found in Table 2). While the percentage of EBV- and CMV-infected individuals did not significantly differ across the three age groups, most of the children in the oldest age group were VZV carriers (Fig. 2B, *P < *0.05 between 1 to 3 and 11 to 17 years of age). Out of the three examined viruses, EBV carriage appeared to impact the proportion of several of the T cell populations examined, with higher proportions of TSCM and TCM CD4^+^ T cells, and lower TN and higher TSCM, TCM, and TEFF CD8^+^ T cells (Fig. 2A). The percentage of the TN subset among CD4^+^ T cells was markedly higher in VZV^–^ compared to VZV^+^ subjects, with an opposite relation for the TCM subset among both CD4^+^ and CD8^+^ T cells (Fig. 2B). We performed quantile regression analysis to further investigate the impact of each virus on T cell composition, either including virus carrier status only in an unadjusted model (data not shown) or including virus carrier status, age, and treatment as covariates (Coef^adj^). This analysis suggests that EBV carriage is associated with the percentage of the TN subset in the CD8^+^ T cell lineage (Table 3; CD8^+^ TN: Coef^adj^ −11.8, *P = *0.020). CMV carriage was associated with the CD4^+^ TN subset (Coef^adj^ 9.8, *P = *0.019).

**TABLE 2:** Subject data

Clinical characteristics	Age 1 to 3 yrs (*n* = 10)	Age 4 to 10 yrs (*n* = 14)	Age 11 to 17 yrs (*n* = 8)	Total (32)	
Autoimmune diagnosis +/− treatment [*n* (%)]					
No treatment	0 (0.0)	5 (35.7)	2 (25.0)	7 (21.9)
MTX only	0 (0.0)	0 (0.0)	1 (12.5)	1 (3.1)
Biological	1 (10.0)	1 (7.1)	1 (12.5)	3 (9.4)
MTX + Biological	0 (0.0)	2 (14.3)	2 (25.0)	4 (12.5)
Serostatus [*n* (%)]					
CMV+	7 (70.0)	7 (50.0)	5 (62.5)	19 (59.4)
VZV+	3 (30.0)	9 (64.3)	7 (87.5)	19 (59.4)
EBV+	3 (30.0)	4 (28.6)	3 (37.5)	10 (31.3)
CMV-VZV-EBV−	0 (0.0)	1 (7.1)	0 (0.0)	1 (3.1)
Seropositive [*n* (%)]					
1 herpesvirus	7 (70.0)	7 (50.0)	3 (37.5)	17 (53.1)
2–3 herpesviruses	3 (30.0)	6 (42.9)	5 (62.5)	14 (43.8)
Seropositive for single herpesvirus [*n* (%)]					
CMV	4 (40.0)	3 (21.4)	1 (12.5)	8 (25.0)
VZV	1 (10.0)	4 (28.6)	2 (25.0)	7 (2.9)
EBV	2 (20.0)	0 (0.0)	0 (0.0)	2 (6.3)
Seropositive for multiple herpesviruses [*n* (%)]					
CMV, VZV	2 (20.0)	2 (14.3)	2 (25.0)	6 (18.8)
CMV, EBV	1 (10.0)	1 (7.1)	0 (0.0)	2 (6.3)
VZV, EBV	0 (0.0)	2 (14.3)	1 (12.5)	3 (9.4)
CMV, VZV, EBV	0 (0.0)	1 (7.1)	2 (25.0)	3 (9.4)

**TABLE 3:** Median quantile regression results from T cell phenotypes and EBV, CMV, or VZV carrier status^*a*^

	EBV+		CMV+		VZV+		>One Virus	
Cell variable	Coef^adj^ (CI_L_, CI_U_)	*P* ^adj^	Coef^adj^ (CI_L_, CI_U_)	*P* ^adj^	Coef^adj^ (CI_L_, CI_U_)	*P* ^adj^	Coef^adj^ (CI_L_, CI_U_)	*P* ^adj^
%CD3	8.1 (−5.5, 21.7)	0.231	−7.2 (−22.5, 8.1)	0.340	10.2 (−5.4, 25.9)	0.188	5.4 (−9.2, 20.0)	0.453
%CD4	5.9 (−4.2, 16.1)	0.239	−0.2 (−13.4, 13.1)	0.978	3.4 (−8.2, 14.9)	0.554	6.1 (−3.3, 15.5)	0.190
%CD8	−3.6 (−10.8, 3.7)	0.318	1.0 (−11.0, 12.9)	0.868	−2.5 (−13.1, 8.2)	0.637	−3.7 (−11.2, 3.9)	0.326
%CD4 TN	−6.7 (−15.5, 2.1)	0.131	9.8 (1.8, 17.8)	**0.019** ^*b*^	−0.8 (−11.9, 10.4)	0.886	−2.1 (−10.2, 6.0)	0.604
%CD4 TSCM	1.3 (−0.6, 3.3)	0.159	0.2 (−1.5, 1.8)	0.857	−0.1 (−2.0, 1.8)	0.920	1.0 (−0.6, 2.6)	0.196
%CD4 TCM	3.9 (−0.6, 8.5)	0.087	−2.0 (−6.9, 3.0)	0.417	0.7 (−4.4, 5.9)	0.769	1.8 (−2.5, 6.1)	0.397
%CD4 TEM	0.2 (−3.6, 4.1)	0.915	−0.0 (−4.7, 4.7)	0.985	−0.1 (−5.7, 5.5)	0.964	0.1 (−3.9, 4.1)	0.967
%CD4 TEFF	0.1 (−0.2, 0.4)	0.328	0.1 (−0.2, 0.5)	0.420	0.0 (−0.4, 0.5)	0.933	0.1 (−0.2, 0.4)	0.392
%CD8 TN	−11.8 (−21.6, 2.1)	**0.020**	−1.1 (−21.6, 19.3)	0.910	2.3 (−15.7, 20.3)	0.795	−3.6 (−18.9, 11.7)	0.632
%CD8 TSCM	2.3 (−1.7, 6.2)	0.244	−1.0 (−5.8, 3.8)	0.673	0.7 (−5.7, 7.1)	0.829	1.7 (−3.3, 6.8)	0.481
%CD8 TCM	1.0 (−0.6, 2.6)	0.203	−0.5 (−2.5, 1.4)	0.591	0.3 (−2.4, 3.0)	0.825	0.5 (−1.6, 2.5)	0.650
%CD8 TEM	−0.5 (−8.4, 7.5)	0.906	−0.8 (−5.8, 4.1)	0.727	−1.0 (−6.0, 4.1)	0.694	−0.8 (−5.0, 3.4)	0.694
%CD8 TEFF	1.8 (−2.5, 6.0)	0.400	1.4 (−1.5, 4.3)	0.319	−0.4 (−3.2, 2.4)	0.783	1.2 (−3.9, 6.3)	0.641

a Coef (CI_L_, CI_U_), coefficient with 95% confidence intervals (L = lower, U = upper); ^adj^, model includes the covariates disease and treatment as binary variables and age as continuous variable.

b Bold numbers indicate significant values.

We did not observe a significant difference in the percentages of carriers of single or multiple herpesviruses across the age categories (Fig. 2C). The carriage with multiple herpesviruses affected the composition of T cell subsets, and we found that TSCM T cells were more abundant in donors with 2 or 3 of the investigated herpesviruses compared to those having a single infection (Fig. 2C). Furthermore, smaller TN and larger TCM and TEFF subsets were noted in the CD8^+^ T cells lineage in donors with 2 or 3 herpesviruses (Fig. 2C). However, these differences were not observed in the adjusted quantile regression analysis (Table 3).

### The size of bulk IFN-γ, IL-17A, and IL-22, although not IL-10, producing T cells increases with age upon polyclonal activation.

**FIG 3:**
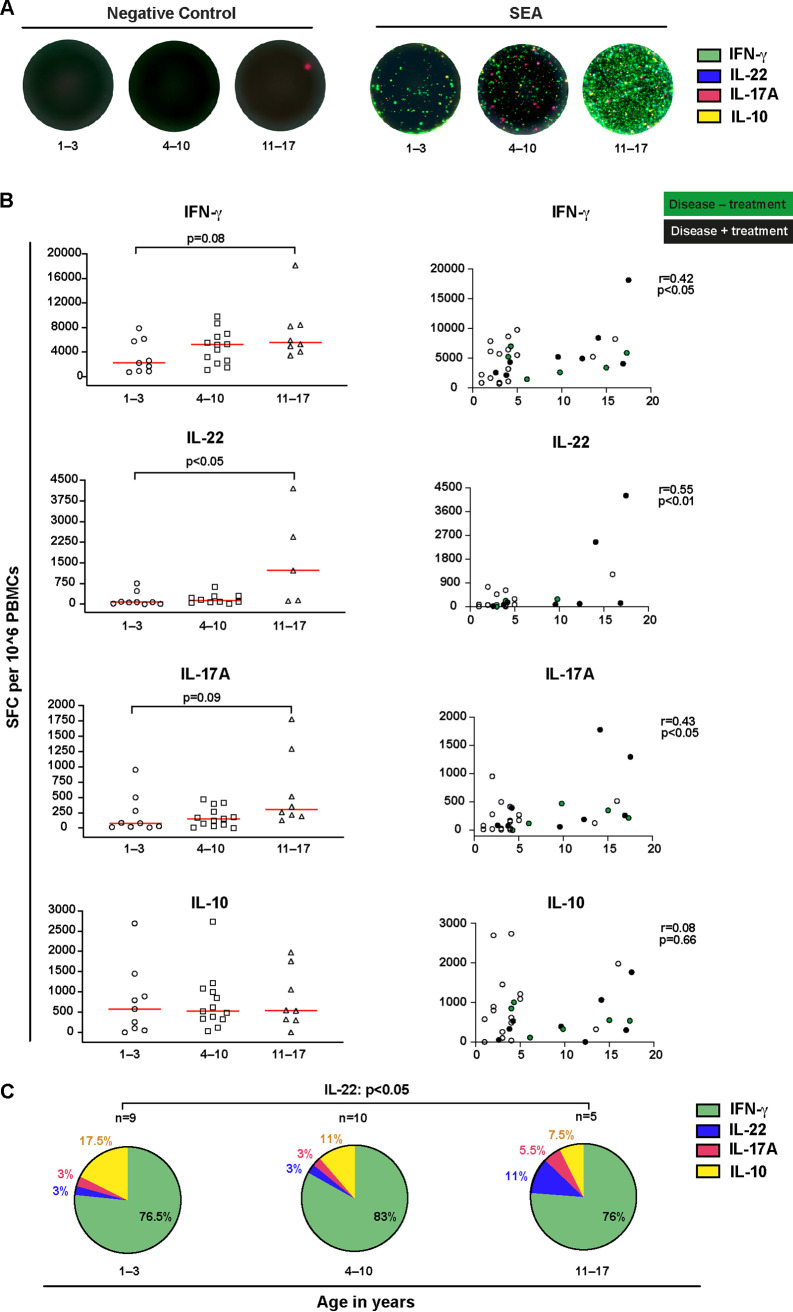
Bulk T cell cytokine responses in children and adolescents latently infected with EBV, VZV, or CMV following a 4-parameter FluoroSpot assay. (A) The impact of age and herpesvirus carriage on T cell cytokine production was examined among children who were 1 to 3, 4 to 10, or 11 to 17 years of age (*n *= 24 for IL-22, *n *= 30 for the remaining cytokines) by a 4-parameter FluoroSpot assay (SFCs, representative image). (B) SFC numbers after polyclonal activation of T cells with the T cell mitogen SEA (left hand graphs) and their correlation with age (right-hand graphs), as tested by Spearman’s rank test. Data from donors with pediatric rheumatic disease on treatment (*n *= 8) are visualized with filled black symbols, and data from those with disease but without treatment (*n *= 6) are visualized with filled green symbols. (C) Compiled profile of the representation of IFN-γ, IL-10, IL-17A, and IL-22 SFCs within the total pool of SFCs of each donor (*n *= 24 donors with data for all four cytokines). Kruskal-Wallis ANOVA was applied to test the statistical differences between the three age categories, and statistical differences were considered if *P* values were at <0.05 after Dunn’s correction for multiple comparisons.

In light of the above results and the fact that memory T cell generation mainly occurs after antigen exposure during infancy, childhood, and young adulthood (6), we went on to examine how age and herpesvirus carriage impact T cell cytokine-producing capacity. We used a 4-parameter FluoroSpot assay, which simultaneously measures IFN-γ-, IL-10-, IL-17A-, and IL-22-secreting cells (Fig. 3A, representative pictures). Upon measurement of SFC numbers after polyclonal activation of T cells with staphylococcal enterotoxin A (SEA), we noticed higher numbers of IL-22 SFC in the oldest group of children compared to the youngest age group (left-hand panel, Fig. 3B, *P < *0.05). We observed a similar pattern for IFN-γ and IL-17A, but without statistical significance (Fig. 3B). When age was used as a continuous variable, positive correlations were found for IFN-γ, IL-22, and IL-17A (right-hand panels, Fig. 3B). Notably, the number of IL-10-producing cells was not associated with age (Fig. 3B). There was no observable clustering of SFC data for the children with autoimmune disease or treatment (filled circles, right-hand panels, Fig. 3B). In addition, virus carriage status did not influence cytokine-producing capacity upon SEA-stimulation.

Next, the relationship between age and the individual T cell cytokine profile was evaluated by measuring the proportion of each cytokine within the total number of SFCs detected (Fig. 3C). Children in the oldest age group had higher proportions of IL-22 SFCs following polyclonal T cell activation compared to the youngest group of children (median 10.3% [IQR 2.5 to 17.2%] and median 2.7% [IQR 0.6 to 5.7%], respectively) (Fig. 3C; *P < *0.05). A proportional decrease in IL-10 SFCs was paralleled with older age (Fig. 3C), which, however, did not reach statistical significance. To assess if the carriage of one or multiple herpesviruses influenced the spectrum of the IFN-γ-, IL-10-, IL-17A-, and IL-22-producing T cell pool, the age groups were further stratified into single and coinfected, resulting in no marked differences (Fig. S2A).

### VZV peptides induce a significant proportion of IL-10-producing T cells.

**FIG 4:**
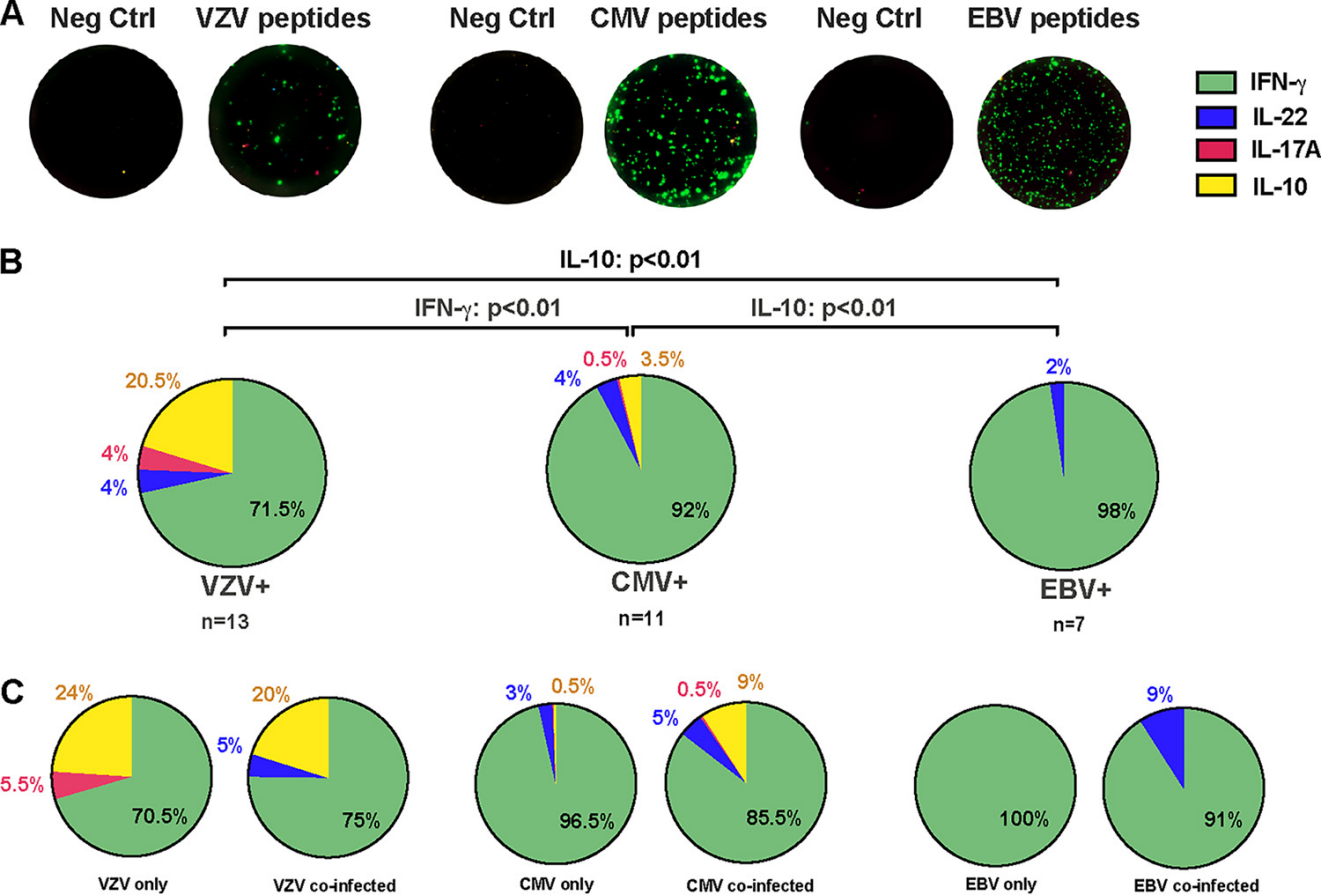
Herpesvirus-specific cytokine responses in children and adolescents latently infected with EBV, VZV, or CMV. (A) T cell cytokine responses to a range of CMV, VZV, and EBV MHC class I and II-restricted peptides were used to trigger virus-specific IFN-γ, IL-17A, IL-22, and IL-10 spot-forming cells (SFCs) in subjects seropositive for each of the respective viruses (representative image). (B) The percentage of SFCs producing either type of cytokine from donors having a complete cytokine data set for IFN-γ, IL-17A, IL-22, and IL-10 SFCs was compared across EBV (*n *= 7), CMV (*n *= 11), and VZV (*n *= 13) seropositive children, regardless of coinfection, disease, treatment, or age. (C) The same three groups (as in B) were further stratified based on whether or not they were infected with multiple herpesviruses. Kruskal-Wallis ANOVA was applied to test the statistical differences among the three age categories, and statistical differences were considered if *P* values were <0.05 after Dunn’s correction for multiple comparisons.

To better understand the contribution of persistent viruses to memory T cell function during childhood, cytokine responses to a range of CMV, VZV, and EBV MHC class I and II-restricted peptides were used to trigger virus-specific IFN-γ, IL-17A, IL-22, and IL-10 SFCs in subjects seropositive for each of the respective viruses (representative pictures, Fig. 4A). The percentage of SFCs producing either type of cytokine from donors having a complete cytokine data set was compared across the three groups based on their carriage of each herpesvirus type, regardless of coinfection, disease, treatment, or age (Fig. 4B; *n *= 13 VZV^+^, *n *= 11 CMV^+^, and *n *= 7 EBV^+^).

The virus-specific IFN-γ SFCs dominated in all three groups and were higher in response to CMV compared to VZV peptides (Fig. 4B; *P < *0.01). The percentages of IL-22 SFCs were similar across the three groups, while T cell responses to VZV were characterized by a higher IL-10 SFC population compared to both CMV and EBV (Fig. 4B; *P < *0.01). Also, the percentage of CMV-specific IL-10 SFCs was significantly higher than EBV-specific IL-10 SFCs (Fig. 4B; *P < *0.01). Strikingly, EBV-induced IL-10 and IL-17A SFCs were nearly undetectable in PBMC cultures derived from EBV^+^ children (Fig. 4B). Correlation analysis between age and the number of the four cytokine SFCs was possible for CMV- and VZV-induced responses. For CMV, age was positively associated with IL-17 A SFC (*r *= 0.62, *P = *0.017), while for VZV, age was negatively associated with IL-22 (r = −0.59, *P = *0.037) and tended to be positively associated with IFN-γ (Fig. S2B and C). Again, no clear clustering of data was found for subjects with disease or treatment (filled circles, Fig. S2B and 2C). Stratification of the VZV-, CMV-, and EBV-infected groups based on carriage of single or multiple herpesviruses revealed no significant differences upon pairwise comparisons by means of SFC percentages. Although small groups, we observed slightly higher diversity in cytokine responses to CMV and EBV in coinfected children (Fig. 4C). For VZV-infected subjects, IL-10 SFCs constituted a noticeable proportion of the total SFCs, together with either IL-17A or IL-22, in VZV single- and coinfected children, respectively (Fig. 4C). Of note, VZV peptides induced a markedly higher IL-10 to IFN-γ SFC ratio than that induced by CMV peptides (*P < *0.01), which was not observed for IL-17A or IL-22 (Fig. S3).

## DISCUSSION

Despite the existing body of literature that focuses on the properties of T cell responses against human herpesvirus, there is a clear lack of studies that extend our knowledge beyond most common T cell cytokines IFN-γ, TNF, and IL-2 against herpesviruses. Another aspect here that has received far less attention deals with immunity against herpesviruses during homeostasis, without the impact of severe immune malfunction, immunosuppression, or immunosenescence. So far, research has been rather biased, with most studies conducted in a disease setting, including HIV-1, cancer, or organ transplant, usually in adults or the elderly (18, 22, 23). We chose to focus on EBV, CMV, and VZV responses, as these viruses are well studied and found in sufficient frequencies among children to be able to group them into infected and noninfected (24, 25). In addition, EBV, CMV, and VZV all have clinical implications, as they can cause complications in pediatric oncology and rheumatology patients subjected to immunosuppressive treatment. For instance, EBV and CMV reactivation during bone-marrow transplantation can be life-threatening, while VZV reactivation, which is most common in these patients, can lead to herpes zoster, with a risk of secondary bacterial infection and postponed treatment (26 to 28). We focused on IFN-γ, IL-22, IL-17A, and IL-10 cytokine responses in a multiparametric setup that mirrored a broader composition of memory T cell populations (29) committed to immune control of herpesviruses during homeostasis.

We confirmed that age is the most influential factor in the expanding size of memory T cell subsets, paralleled with a decrease in TN T cell populations. Interestingly, neither PRD nor treatment against it appeared to have any significant effect on the size of the memory T cell subsets. The size of one subset, TN CD4^+^ T cells, was negatively associated with immunosuppressive treatment. PRD patients appear to have alterations in the subset composition of CD4^+^ T cells due to the disease itself, as previously shown (30). However, MTX treatment over a 6-month period was shown to enhance TN CD4^+^ T cells in another study of PRD patients (31). The reason why PRD in our subjects was not associated with altered CD4^+^ T cell populations remains elusive. We have previously studied a larger (*n *= 78) group of children with PRD with respect to their circulating T cell population immunophenotypes (32). In those children, PRD was not associated with any alterations in their classical T cell subsets upon comparison with healthy controls (32). The current study used a subgroup of PRD patients included in our former study (32), and therefore it is reasonable to not expect any major variation in the T cell composition in the PRD group, even if the group size is smaller.

In line with what is currently known (1, 2, 15, 18), dynamics in T cell subsets, especially those within the CD8^+^ lineage, were observed based on herpesvirus carriage. Here, EBV emerged as the most influential virus among the three studied, while fewer populations appeared affected by VZV carrier status, and no differences were seen for CMV. It is somewhat surprising, and in contrast to previously published data, that CMV-driven maturation of memory T cell phenotypes is not evident in our subjects. The duration of virus carriage, which is a central factor in the shaping of memory T cell populations, was not feasible to assess in this study. Considering previous observations in longitudinal pediatric cohorts from us and others, EBV infection seems to have a significant impact on the immune system during early life (1, 2, 12, 24, 25), which is less pronounced than CMV in adulthood. This can in part be due to repeated subclinical reactivation and the emergence of terminally differentiated memory T cells (33).

To our knowledge, this study is among the first to complement immunophenotype data in healthy children, in addition to a group on immunomodulating treatment, with functional data from a variety of cytokine-producing T cells polyclonally and specifically for each of the three investigated herpesviruses. We observed that age was a central factor for increased proportions of IFN-γ-, IL-17A-, and IL-22-producing T cells, while no significant correlation was found between age and IL-10-producing T cells in bulk. Antigen-specific T cell responses to all three viruses were dominated by IFN-γ production, yet a significant proportion of VZV-specific T cells released IL-10, as confirmed following comparison of IL-10^+^/IFN-γ^+^T cell ratios between VZV- and CMV-stimulated PBMCs. Interestingly, clinical data from adult patients with herpes zoster or VZV indicate that high levels of IL-10 can be detected in body fluids at the time of infection and have predictive value for the severity of infection (34, 35).

Interpretation of the results should, however, also be in light of the weaknesses of the study. First, as there is no recommendation for childhood VZV vaccination in Sweden, we have assumed that the children that were seropositive had acquired VZV through natural infection. Another drawback in our study is the inability to determine whether IL-10 originated from CD4^+^ or CD8^+^ T cells. We speculate that CD4^+^ T cells are likely to contribute to this IL-10 response, in line with the earlier study showing that CD4^+^ T cell responses were central in the control of VZV infection (20). Considering this, the role of polyfunctional T cells (36) would be valuable to investigate further. Of note, descriptive studies on similar types of material and readouts are, to our knowledge, missing or incomplete by means of antigen specificity, herpesvirus types, and immune cell type (1, 12, 37, 38).

We believe that this study may contribute to expanding our knowledge about the spectrum of cytokines produced by T cells following encounters with herpesviruses. Our findings show a deviation between polyclonal and herpesvirus-specific T cell responses in children, which needs to be taken into consideration when interpreting the associations between herpesvirus carrier status and bulk T cell reactivity in otherwise healthy hosts. Furthermore, our findings suggest that cytokine responses can vary depending on the type of herpesvirus, which holds true particularly for IL-10-producing T cells following VZV antigen stimulation. In a broader context, our findings may have implications for the immunocompromised host with VZV reactivation, which in turn may drive the expansion of T cell clones that produce a regulatory cytokine that counterbalances the effect of other T cell clones needed to combat malignant cells.

## MATERIALS AND METHODS

### Study subjects, sample processing, and determination of CMV, VZV, and EBV status.

This study was approved by the regional ethical review board in Stockholm (Dnr 2020–05664 and Dnr 2014-1164-31/1), and informed consent was obtained from all the subjects’ parents or legal guardians before collection of blood samples. A total of 32 children between 1 and 17 years of age were included in this study. With respect to knowledge about the dynamics of T cell populations and pathogen susceptibility (6), the subjects were divided into three age groups: 1 to 3 years old, 4 to 10 years old, and 11 to 17 years old (Table 1). A subset of the included children (*n *= 15) was recruited between 2011 and 2014 at the Pediatric Rheumatology Unit at Astrid Lindgren Children’s Hospital, Stockholm, following a diagnosis with PRD as previously described (32, 39). This group contained subjects not under treatment (*n *= 7); subjects under treatment with the immunosuppressant drug methotrexate (MTX) alone (*n *= 1); subjects under treatment with biological therapy (anti-IL-6, anti-TNF, or anti-IL-1) alone (*n *= 3); and subjects treated with MTX in combination with a biological therapy (*n *= 4) (Table 1). The remaining children (*n *= 17) were otherwise healthy and not under treatment with any medication. These were recruited between 2014 and 2016 at the laboratory of Astrid Lindgren Children’s Hospital in conjunction with routine blood sampling for reasons other than acute infections, fever, or immune-mediated diseases.

Following the sampling of peripheral venous blood, plasma was isolated and stored at −80°C, and PBMCs were separated by Ficoll-Hypaque gradient centrifugation (GE Healthcare Bio-Sciences AB, Uppsala, Sweden). The cells were washed and resuspended in freezing medium consisting of 90% FCS (Gibco, Invitrogen, Carlsbad, CA, USA) and 10% dimethyl sulfoxide (Sigma-Aldrich, St. Louis, MO, USA) and stored in liquid nitrogen until downstream analyses. In a subset of children (*n *= 16), IgG seropositivity for CMV and EBV was determined by routine analysis of virus-antigen specific IgG by the hospital’s clinical chemistry laboratory. IgG seropositivity against VZV for all (*n *= 32) subjects and against EBV and CMV for the remaining (*n *= 16) children was determined through measurement of EBV-VCA, CMV, and VZV IgG Platiela-ELISA (Bio-Rad Laboratories, Hercules, CA, USA), in accordance with the manufacturer’s instructions. One child was excluded from further analyses due to seronegativity for all three herpesviruses (Table 1).

### Cell culture conditions and immunophenotyping of T cell subsets by flow cytometry.

PBMCs were thawed, washed, counted with trypan blue staining, resuspended to a concentration of 2 × 10^6^ live cells/mL in cell culture medium (RPMI 1640 supplemented with 10% FCS, l-glutamine [2 mmol/L], penicillin G sodium [100 U/mL], and streptomycin sulfate [100 g/mL], all from Thermo Fisher Scientific), and rested overnight at 37°C and 5% CO_2_. PBMCs were then washed with PBS and incubated with a dead cell marker (DCM, Fixable Aqua Dead Cell Stain kit, Invitrogen) for 10 min at 4°C in the dark, ahead of washing and labeling with fluorescent-conjugated antibodies against CD3, CD4, CD8, CCR7, CD45RO, and CD95. Data were acquired by Novocyte 3000 (ACEA Biosciences) and analyzed using FlowJo software (FlowJo LLC, USA). Gating of CD4^+^ and CD8^+^ T cell subsets was performed according to previously published procedures by Gattinoni et al. (8) by differential expression patterns of CCR7, CD45RO, and CD95. FMO controls were used for CCR7, CD45RO, and CD95 gating. Naïve (TN) CCR7^+^CD45RO^−^CD95^−^; stem-central memory (TSCM) CCR7^+^CD45RO^−^CD95^+^; central memory (TCM) CCR7^+^CD45RO^+^CD95^+^; effector memory (TEM) CCR7^−^CD45RO^+^CD95^+^; and terminal effector (TEFF) CCR7^−^CD45RO^−^CD95^+^ T cells were subsequently defined (Fig. 1A).

### Four-color FluoroSpot of IFN-γ-, IL-22-, IL-10-, and IL-17A-secreting cells.

The number of IFN-γ, IL-22-, IL-10-, and IL-17-A-secreting cells (referred hereafter as spot-forming cells, SFC) were determined with a human IL-22/IFN-γ/IL-10/IL-1-7A FluoroSpot Plus kit (Mabtech AB, Nacka Strand, Sweden) according to the manufacturer’s instructions. Briefly, in sterile conditions throughout the procedure, FluoroSpot plates precoated with cytokine-specific monoclonal antibodies were washed with PBS and thereafter blocked with cell culture medium for 1 h. Following removal of the blocking medium, T cell-activating reagents were added to different wells of the plates. The mitogen staphylococcal enterotoxin A (SEA, final concentration 20 ng/mL, from Staphylococcus aureus, Sigma-Aldrich) served as a polyclonal activator of T cells. Herpesvirus-specific responses to CMV, VZV, and EBV were assessed upon stimulation with mixed peptide pools representing acute and latency MHC class I and II-restricted epitopes. The CMV peptide pool (Mabtech AB) was used at 2 μg/mL final concentration; the VZV PepMix was used at 1 μg/mL final concentration (for each of the IE63 and gE antigens); and the EBV PepMix Collection was used at 1 μg/mL final concentration (both from JPT Peptide Technologies GmbH, Germany). Furthermore, an anti-CD28 monoclonal antibody (Mabtech) was added, according to the manufacturer’s instructions, to all wells for costimulation of T cells. Overnight rested PBMCs were washed with PBS, counted as previously mentioned, and resuspended in fresh cell culture medium.

A total of 100,000 live PBMCs were thereafter transferred to each well for T cell stimulation with SEA or the cell culture medium alone (serving as the corresponding negative control). A total of 300,000 live PBMCs were thereafter transferred to each well for antigen-specific T cell stimulation or to wells containing a cell culture medium supplemented with the peptide pool diluent (serving as a negative control). All stimulations were set in duplicates, and the plate was incubated for 48 h in 37°C, humidified 5% CO_2_ atmosphere. Following cell culture supernatant isolation and storage at −80°C, the FluoroSpot plates were sequentially washed, and reagents for detection of cytokine-producing SFCs were added, in line with the manufacturer’s protocol. Plates were read using the Mabtech IRIS ELISpot/FluoroSpot reader (Mabtech AB) equipped with wavelength-specific filters corresponding to DAPI, FITC, Cy3, and Cy5 spectra for detection of each cytokine separately. Each fluorescent spot represents the secretary footprint of a single cell. The frequency of spots was assessed with Apex software version 1.1 (Mabtech AB). Data in figures are presented as SFCs per 10^6^ PBMCs, in mitogen- or herpesvirus antigen-stimulated wells, after subtraction of SFC in the background control wells.

### Enzyme-linked immunosorbent assay (ELISA) for IL-6 detection in cell culture supernatants.

CMV, EBV, and VZV peptide libraries were controlled for contamination by endotoxins through measurement of IL-6 in cell culture supernatants from four donors with differential CMV, EBV, and VZV carrier statuses using the human IL-6 ELISA development kit (Mabtech AB). The IL-6 levels were either below the detection limit for 2 donors or at the borderline of the lowest detection limit in the remaining 2 donors (data not shown).

### Statistics.

Nonparametric Kruskal–Wallis ANOVA was applied to test the statistical differences between three independent groups, and statistical differences were considered if *P* values were at <0.05 after Dunn’s correction for multiple comparisons. The Mann-Whitney U test was used to compare the two independent groups. Spearman’s rank correlation was used to test for associations between the two continuous variables. Associations between the percentage of naive and memory T cell subsets and clinical parameters (age, disease, and treatment) or virus status were investigated unadjusted or adjusted (with the clinical parameters in the model) median quantile regression models, with data presented as coefficients (coef) and 95% confidence intervals. Lines in scatterplots represent the median. The data described in the Results section depict median values and interquartile range (IQR). Percentages of each cytokine-producing cell among total cytokine-producing cells were calculated for those donors from whom complete data on the four examined cytokines were available. Following this, pie charts were plotted on median values for each group, which were used for statistical testing with the Kruskal-Wallis ANOVA, as mentioned before. Analyses were carried out using Prism (v.9.1.0, GraphPad Software, LLC, San Diego, USA) and Stata (version 16.1, StataCorp LP, College Station, TX, USA).
