# Supplementary material for: Four-Parameter FluoroSpot Assay Reveals That the Varicella Zoster Virus Elicits a Robust Memory T Cell IL-10 Response throughout Childhood
Source: J Virol. 2022 Oct 31;96(22):e01310-22. doi: 10.1128/jvi.01310-22 (PMC9683015; doi:10.1128/jvi.01310-22)
Supplement: Supplemental file 1 — Fig. S1 to S3. Download jvi.01310-22-s0001.pdf, PDF file, 0.2 MB [file jvi.01310-22-s0001.pdf]

SUPPLEMENTAL FIGURE 1

Disease – treatment
Disease + treatment

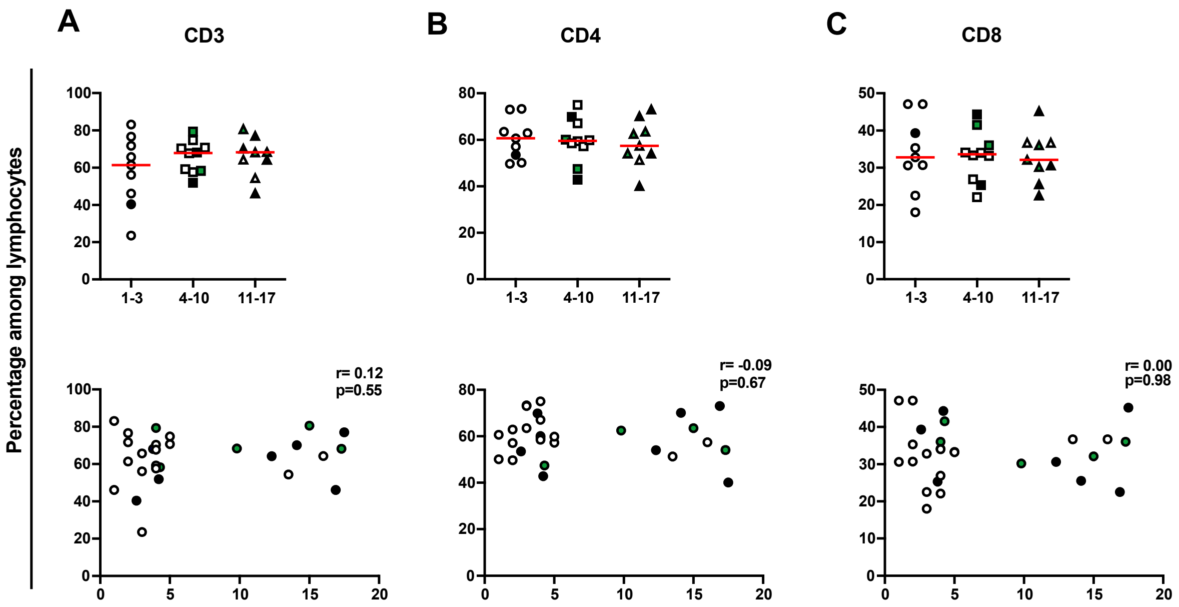

Supplemental Figure 1. Relationship between age and bulk T cells in healthy children and children with autoimmune disease. (**A**) Proportions of $CD3^+$ lymphocytes or **(B)** $CD4^+$ and **(C)** $CD8^+$ cells derived from the $CD3^+$ lymphocyte gate originating from peripheral blood mononuclear cells derived from children grouped in three age categories (n = 28), among whom a subset (n = 12) had pediatric rheumatic disease, with (n = 7, filled black symbols) or without treatment (n = 5, filled green symbols). Correlation between age and proportions of $CD3^+$ cells $CD4^+$ T cells and $CD8^+$ T cells (bottom graphs) were tested by Spearman's rank test. Kruskal–Wallis ANOVA was applied to test the statistical differences between the three age categories, and statistical differences were considered if p-values were < 0.05 after Dunn's correction for multiple comparisons.

SUPPLEMENTAL FIGURE 2

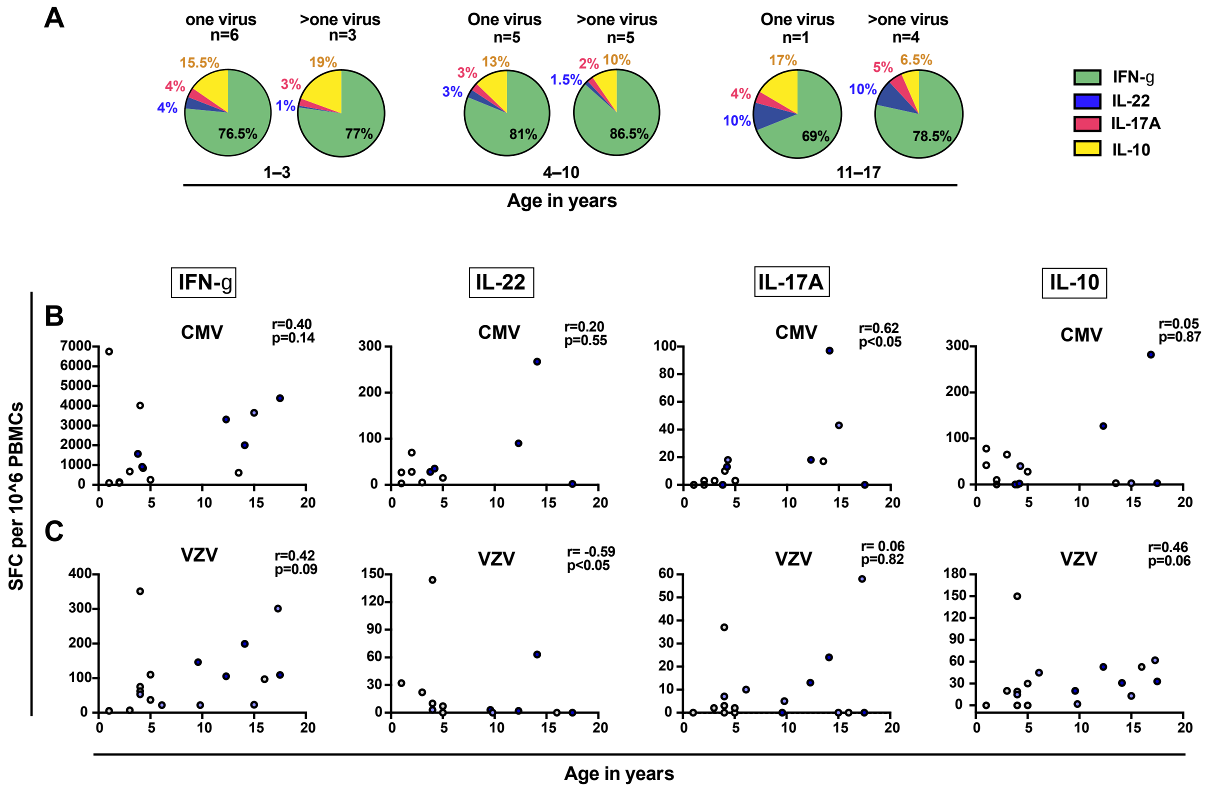

Supplemental Figure 2. Influence of one or multiple herpesviruses on the spectrum of IFN-γ, IL-10, IL-17A, and IL-22 producing T cell pools. (**A**) Children grouped into three age groups were further stratified based on having single and co-infection with herpesviruses EBV, CMV, or VZV. **(B, C)** Correlation analysis between age and the number of the IFN-γ, IL-17A, IL-22, and IL-10 spot forming cells (SCFs) after CMV (**B**) or VZV (**C**) peptide-pool stimulation of peripheral blood mononuclear cells from healthy children (white symbols) or children with pediatric rheumatic disease with (filled dark blue symbols) and without (filled light blue symbols) treatment. Correlations between age and SFCs were tested using Spearman's rank test.

SUPPLEMENTAL FIGURE 3

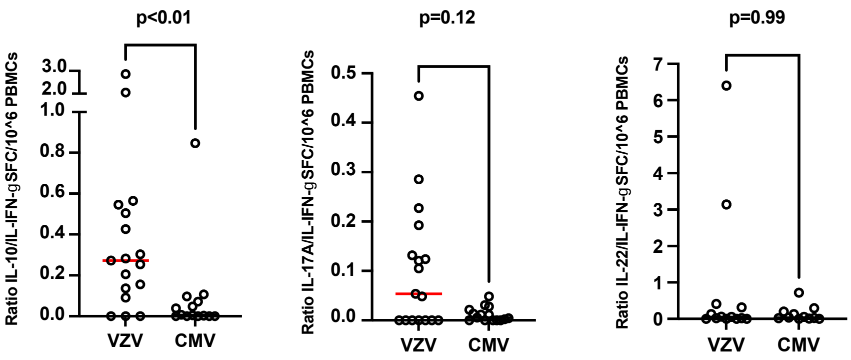

Supplemental Figure 3. IL-10, IFN-γ , IL-17 A, and IL-22 spot forming T cell ratios between VZV⁺ and CMV⁺ children. Comparison of IL-10/ IFN-γ , IL-17A/IFN-γ, and IL-22/IFN-γ spot forming T cell ratios between VZV⁺ (n=17) and CMV⁺ (n=15) children. The Mann–Whitney U-test was used to compare two independent groups.
